# *PDCD10* Gene Mutations in Multiple Cerebral Cavernous Malformations

**DOI:** 10.1371/journal.pone.0110438

**Published:** 2014-10-29

**Authors:** Maria Sole Cigoli, Francesca Avemaria, Stefano De Benedetti, Giovanni P. Gesu, Lucio Giordano Accorsi, Stefano Parmigiani, Maria Franca Corona, Valeria Capra, Andrea Mosca, Simona Giovannini, Francesca Notturno, Fausta Ciccocioppo, Lilia Volpi, Margherita Estienne, Giuseppe De Michele, Antonella Antenora, Leda Bilo, Antonietta Tavoni, Nelia Zamponi, Enrico Alfei, Giovanni Baranello, Daria Riva, Silvana Penco

**Affiliations:** 1 Department of Laboratory Medicine, Medical Genetics Unit - Niguarda Ca' Granda Hospital, Milan, Italy; 2 Regional Centre for Epilepsy of Childhood and Adolescence - Children's Hospital of Brescia, Brescia, Italy; 3 Paediatrics & Neonatology Unit, Eastern Liguria Hospital, La Spezia, Italy; 4 Neurosurgery Unit, G.Gaslini Institute, Genova, Italy; 5 Department of Pathophysiology and Transplantation, University of Milan, Milan, Italy; 6 Child Neurology and Psychiatry Unit - AUSL Bologna, Bologna, Italy; 7 Department of Human Motor Sciences and Neuromuscular Diseases Unit, Institute of Aging, “G. D'Annunzio” University, Chieti-Pescara, Italy; 8 Neurology Unit- AUSL Bologna - Bellaria Pizzardi Hospital, Bologna, Italy; 9 Foundation IRCCS Neurological Institute Carlo Besta, Milan, Italy; 10 Neurosciences and Reproductive and Odontostomatological Sciences, Federico II University, Naples, Italy; 11 Child Neuropsychiatry - A.O.U United Hospitals of Ancona, Ancona, Italy; Pasteur Institute of Lille, France

## Abstract

Cerebral cavernous malformations (CCMs) are vascular abnormalities that may cause seizures, intracerebral haemorrhages, and focal neurological deficits. Familial form shows an autosomal dominant pattern of inheritance with incomplete penetrance and variable clinical expression. Three genes have been identified causing familial CCM: *KRIT1*/CCM1, *MGC4607*/CCM2, and *PDCD10*/CCM3. Aim of this study is to report additional *PDCD10*/CCM3 families poorly described so far which account for 10-15% of hereditary cerebral cavernous malformations. Our group investigated 87 consecutive Italian affected individuals (*i.e.* positive Magnetic Resonance Imaging) with multiple/familial CCM through direct sequencing and Multiplex Ligation-Dependent Probe Amplification (MLPA) analysis. We identified mutations in over 97.7% of cases, and *PDCD10*/CCM3 accounts for 13.1%. *PDCD10*/CCM3 molecular screening revealed four already known mutations and four novel ones. The mutated patients show an earlier onset of clinical manifestations as compared to CCM1/CCM2 mutated patients. The study of further families carrying mutations in *PDCD10*/CCM3 may help define a possible correlation between genotype and phenotype; an accurate clinical follow up of the subjects would help define more precisely whether mutations in *PDCD10*/CCM3 lead to a characteristic phenotype.

## Introduction

Cerebral cavernous malformation (CCM; OMIM 116860) is one of the most common types of vascular malformations characterized by “blackberry-like” aggregation of grossly enlarged capillary cavities consisting of a single layer of endothelium without intervening neuronal tissue [1].

Cavernous malformations can occur anywhere in the body - brainstem, cerebellum, spinal cord, cranial nerves, cerebral ventricles, retina, skin and liver - but are most commonly found in the forebrain [2]. They occur as single or multiple lesions and, depending on size and location, can be clinically silent or show clinical symptoms ranging from headache to focal neurological deficits, seizures and fatal intra-cerebral haemorrhage [3]–[5].

CCM can arise in a sporadic form, with a single lesion, or in a familial form, with multiple cavernous malformations [6]. The familial form shows an autosomal dominant pattern of inheritance with incomplete penetrance and variable clinical expression [7]. However, multiple lesions have been found in patients with no positive family history [8] and combined clinical and genetic tests have recently revealed that the vast majority of these ‘sporadic cases’ with multiple lesions have indeed a genetic origin: a *de novo* mutation or a mutation inherited from an asymptomatic parent [2], [9]–[11].

Genetic studies identified three CCM genes in different loci: *KRIT1*/CCM1 at 7q21–22 [12], [13], *MGC4607*/CCM2 at 7p13–15 [14], [15] and *PDCD10*/CCM3 at 3q25.2–27 [16], [17].

Several mutations have been identified so far in the Italian population, all of them appearing to cause a loss of function [15], [18]–[26].Starting from 2004 we have investigated 87 consecutive index cases, with the presence of multiple angiomas and/or with a positive family history (FCCM); we identified mutations in over 97.7% of FCCM cases Among the positive cases, *KRIT1*/CCM1 accounts for 68.9%; *MGC4607*/CCM2 for 18.0% and *PDCD10*/CCM3 for 13.1%.

Our group identified mutations in over 97.7% of FCCM cases, this high score of mutation detection being due to the selection of index case according to the presence of multiple angiomas and/or to a positive family history (FCCM). Not surprisingly, high prevalence of causative mutation has been identified in *KRIT1*/CCM1 followed by *MGC4607*/CCM2 and *PDCD10*/CCM3, these last two with quite similar mutation rate.

According to recent data *PDCD10*/CCM3 mutations cause 10–15% of FCCM [5] and less than 40 CCM3 families have been reported so far [15], [17], [19], [27]–[31]. This limited number of patients harbouring a mutation in *PDCD10*/CCM3 gene hampered establishing the genotype-phenotype correlations. It has been recently reported that *PDCD10*/CCM3 mutation carriers display earlier symptoms' onset, usually before 15 years of age, and higher risk of cerebral haemorrhage during childhood; multiple meningiomas are frequently reported too. However the mechanisms leading from CCM3 mutations to meningiomas are still unknown [5], [28], [29].

We report here the results related to *PDCD10*/CCM3 gene molecular screening carried out on eleven unrelated Italian CCM affected patients, all of them were found to harbour mutations, four already known and four novel ones.

## Results

**Figure 1:**
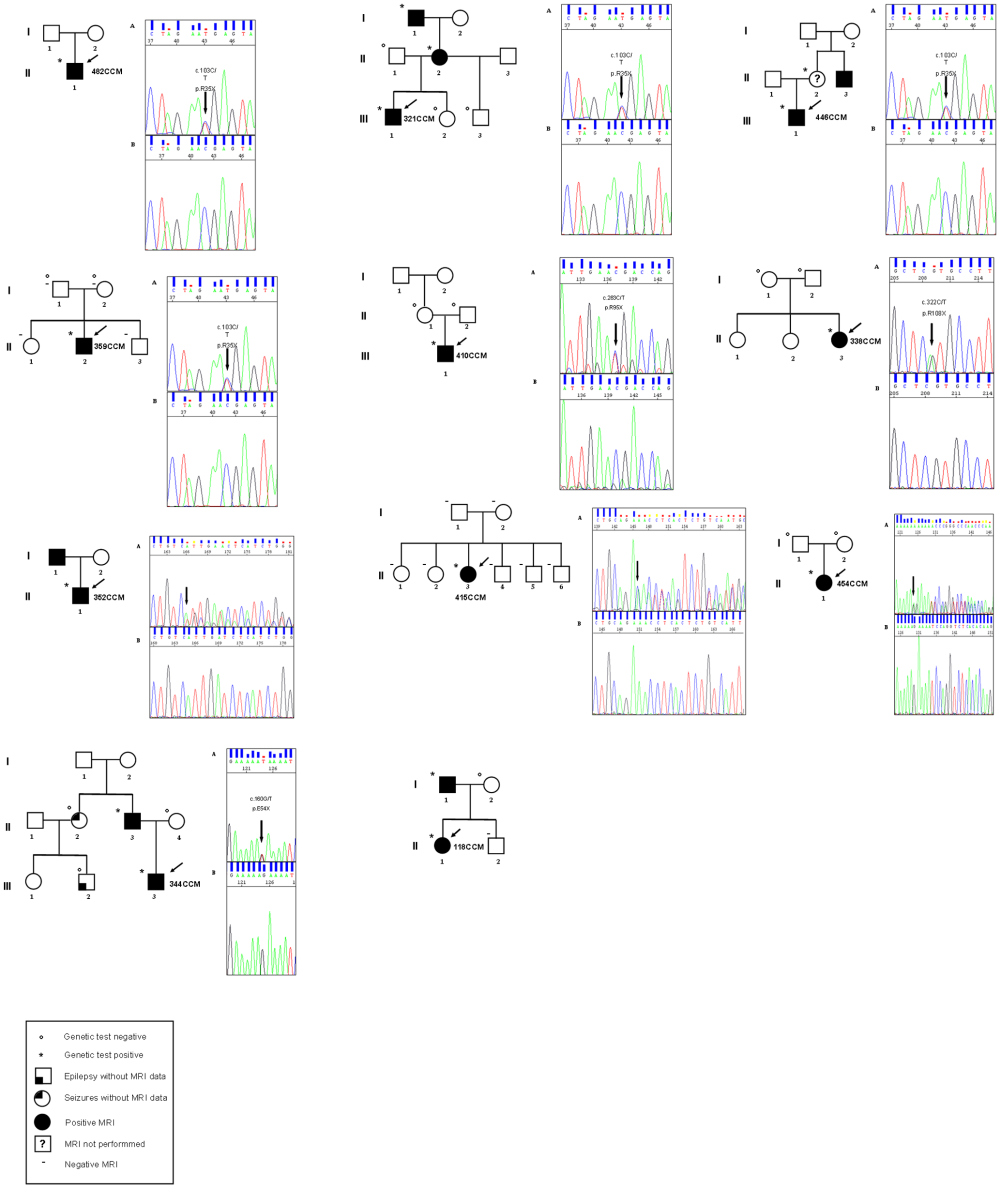
Pedigrees of the 11 patient harbouring a *PDCD10*/CCM3 mutation. The arrow indicates the index case. Squares represent males; circles, females. A diagonal line through the symbol represents a deceased person.

We analyzed 87 Italian cases with multiple lesions and/or positive family history; 11 index patients (13.1%) resulted to be mutated in *PDCD10*/CCM3 in (Fig. 1). When relatives' DNA samples were available, we observed a complete cosegregation of the mutational event with the clinically affected status (i.e., positive MRI).

The eight identified mutations are summarized in Table 1: R35X, R95X, R108X, and a whole gene deletion were already reported in literature [5], [15], [17], [27], [30], while four more mutations are described here for the first time (c.367_387dup; [c.376_380del; 392_393insGACAGAGTGTCTGCAGACTTGATTGTCTGCAGACAAT]; c.159dup and c.160G>T). Among the new mutations, the first one duplicates six aminoacids, the last two introduce a stop codon, while the effect of the remaining one is unknown. The molecular analysis revealed the typical pathogenic loss-of-function mutations with the exception of one duplication, the complex rearrangement and the loss of the entire allele. As for the last one, we were able to observe the cosegregation of the deletion within the affected family members (proband's father, see Tables 1 and 2).

**Table 1:** Details of Patients' mutation.

FAMILY NUMBER	EXON	MUTATION (NM_007217)	THEORETICAL EFFECT ON THE PROTEIN	FAMILY HISTORY	REF	ITALIAN GEOGRAPHICAL ORIGIN
**482 CCM**	5	c.103C>T	R35X	N	Lee et al., 2008	South
**321 CCM**	5	c.103C>T	R35X	Y	Lee et al., 2008	Centre
**321 CCM's mother**	5	c.103C>T				
**321 CCM's grandfather**	5	c.103C>T				
**446 CCM**	5	c.103C>T	R35X	Y	Lee et al., 2008	North
**446 CCM's mother**		c.103C>T				
**359 CCM**	5	c.103C>T	R35X	*de novo*	Lee et al., 2008	Centre
**410 CCM**	7	c.283C>T	R95X	*de novo*	Guclu et al., 2005 Verlaan et al., 2005	North/South
**338CCM**	7	c.322C>T	R108X	*de novo*	Riant et al., 2013	North
**352 CCM**	7	c.367_387dup	D123_Q129dup	Y	novel	Centre
**415 CCM**	7	c.376_380del; 392_393insGACAGAGTGTCTGCAGACTTGATTGTCTGCAGACAAT	p.?	?	novel	South
**454 CCM**	6	c.159dup	E54Rfs*22	*de novo*	novel	Centre
**344 CCM**	6	c.160G>T	E54X	Y	novel	South
**344CCM's father**	6	c.160G>T				
**118 CCM**		whole gene deletion		Y	Liquori et al., 2008	North
**118 CCM's father**		whole gene deletion				

Y =  Yes; N = No. Nomenclature according to HGVS.

**Table 2:** Patients' clinical features.

FAMILY NUMBER	CEREBRAL MR IMAGING	AGE AT ONSET (years)	AGE AT FIRST VISIT (years)	INAUGURAL MANIFESTATION	BLEEDING EVENTS UP TODAY (n°)	NEUROSURGERY		ASSOCIATED CAVERNOMAS			OTHER
						Y/N	n° INTERVENTION	CUTANEOUS	RETINAL	SPINAL	
**482 CCM**	multiple lesions	13	32	involuntary movements at right upper limb	1	N	0	angiokeratomas	N	N	right vocal cord paralysis
**321 CCM**	multiple lesions	1.2	4	mild psychomotor retardation with lack of independent ambulation	nr	N	0	plain angioma on the posterior part of neck	N	N	left facial hypoplasia with asymmetric palate and dental arcades
**321 CCM's mother**	multiple lesions		36	asymptomatic	nr	N	0	2 plain angiomas in the lumbar and abdominal areas, respectively + 2 pinkie hyperkeratotic cutaneous capillary venous malformations (HCCVM) in relief and hairy	N	N	mild left facial hypoplasia
**321 CCM's grandfather**	multiple lesions	30	76	headache	nr	N	0	plain angioma in the neck	N	N	mild facial asymmetry
**446 CCM**	multiple lesions	4 months	31	strabismus, exophthalmos, ptosis, at 1.5 years acute palsy of right 3^rd^ cranial nerve	1	N	0	N	N	N	nr
**446 CCM's mother**	not performed		48		nr	N	0	N	N	N	one brother with CCM lesions underwent surgical intervention
**359 CCM**	multiple lesions	6	13	attention disorder and seizures	N	Y	1	N	ni	ni	nr
**410 CCM**	multiple lesions	8	13	headache	N	N	0	three median cervico-dorsal angiomas	N	isolated medullar cavernous angioma	nr
**338CCM**	multiple lesions	4	4	rigor nucalis, pain during flexion of the neck, hypotonia, difficulties in speech	nr	N	0	N	N	N	occipital scale elevated with crowning of the foramen magnum and cerebellar tonsillar hernia (Chiari I anomaly)
**352 CCM**	multiple lesions	35	39	2 episodes of dysarthria and paresthesias in the right upper limb and ipsilateral hemiface	N	N	0	N	N	N	skull of left parietal region showed impaired signal and contrast enhancement consistent with small bone angioma
**415 CCM**	multiple lesions	1	11	seizures	4	Y	1	N	N	N	nr
**454 CCM**	multiple lesions	1.4	5	palsy of 7th cranial nerve and left hemiplegia	4	Y	1	N	N	N	nr
**344 CCM**	multiple lesions	8	13	left-sided focal sensory-motor seizures, transient hemiparesis and dysarthria	several	Y	1	N	N	N	nr
**344CCM's father**	multiple lesions		42	asymptomatic	nr	N	0	N	ni	ni	One sister and one nephew are reported to suffer from idiopathic epilepsy
**118 CCM**	multiple lesions	2	11	bleeding cavernous angioma at the pontine site	3	Y	2	N	N	N	growth retardation
**118 CCM's father**	multiple lesions	15	42	seizures	N	N	0	N	N	N	nr

Y = Yes; N = No; nr =  not reported; ni =  not investigated.

Pathogenicity of the new mutations was established through predictive software such as MutationTaster and Mutalyzer [32], [33].

Four cases (338CCM, 359CCM, 410CCM and 454CCM) resulted to be *de novo* mutations since both parents did not harbour the mutation itself. DNA profiling using STR multiplex assay was applied to the probands and their parents to determine and confirm their relationship: paternity was established in all the four cases.

As regards 415CCM subject, we were not able to demonstrate the *de novo* nature of the mutation since DNA samples from parents were not available. Proband's siblings, ranging from 3 to 14 years and parents, both aged 41 years, were negative at MRI study.

As for 344CCM subject, surprisingly we did not find the mutation in two family members both presenting epilepsy (II;2 and III;2). Apparently the proband's father (III;3) harbouring the mutation doesn't present any symptom, but cerebral MRI resulted positive for the presence of cavernomas. The proband's grandparents were referred to be in healthy status.

Among the seven familial cases, inheritance appears to be paternally derived four times (321CCM, 352CCM, 344CCM and 118CCM) while we observed maternal origin only in one case (446CCM); in two cases (482CCM and 415CCM), we were not able to assess the inheritance pattern.

Prenatal diagnosis was requested from 118CCM's family: after an accurate genetic counselling it has been performed through MLPA with flanking exon probes, giving a negative result. This has been confirmed later on the newborn DNA (II;2).

**Figure 2:**
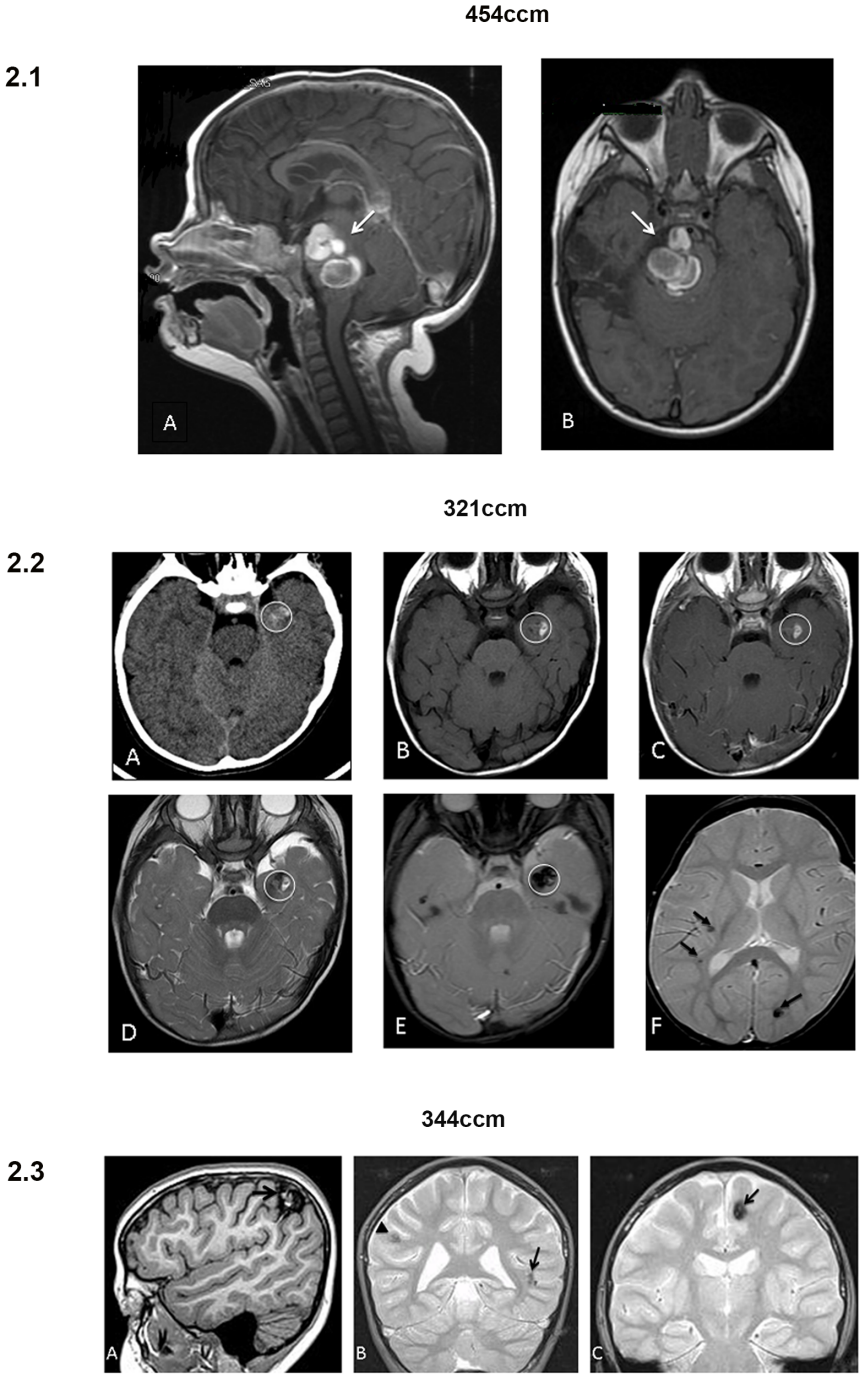
1–3 MRI/TC scans from subjects : 1) 454CCM patient harbouring the *de novo* and novel mutation p.E54Rfs*22. A) T1 sagittal image at 16 months showing a cavernous malformation with recent bleeding in the pons; B) T1 axial image at 25 months showing increased size of the pontine cavernous malformation with compression on the mesencephalon, the cisterna interpeduncularis and the cisterna pontis. 2) 321CCM patient harbouring the mutation p.R35X. A) Imaging characteristics of the CCM lesion (in the white circle) located at the left anterior temporal lobe: at CT scan the lesion is inhomogeneous due to haemorrhagic components, B and D) the haemorrhagic component is hyperintense both in T1 and in T2 sequences, C) contrast enhancement is absent, E) and at GET2* the lesion is hypointense due to the paramagnetic characteristics of the haemosiderin ring and of the clotted lesion content. F) Finally, other two lesions can be detected at other sites (arrows) in the same patient. 3) 344CCM patient harbouring the novel mutation p.R54X. A) MRI showed a right cortical and subcortical parietal hemorrhagic CCM lesion (arrow) and other non-hemorrhagic CCM lesions at different sites: bilateral temporal polar (not shown), B) left superior temporal sulcus (arrow) and right parietal (arrowhead), left insular and fronto-insular (not shown), C) left frontal parasagittal (arrow), subcortical frontal with small areas of vacuolization and microcalcification, left posteromedial thalamic (not shown).

In all the tested subjects no associations were found with other cerebral vascular malformations, such as meningiomas or venous cavernomas or arterovenous malformations,. The mean age of the first onset is 7.3 years (range 0.33–35 years) and in 5 out of 11 patients we found extra-axial cavernous angiomas. Clinical characteristics of patients are summarized in Table 2, while MRI/TC imaging of patients 321CCM, 344CCM and 454CCM are reported in Figure 2.

## Discussion

The CCM3 gene [Programmed cell death 10 (*PDCD10*)] is highly conserved in both vertebrates and invertebrates and is the most recently discovered compared with CCM1 and CCM2 [16], [17]. It has been shown that PDCD10 interacts in vitro with the other two proteins involved in genesis of cavernomas: K-Rev interaction trapped 1 (KRIT1) and Malcavernin, which participates in CCM1-dependent modulation of β1-integrin-mediated signalling and CCM2-mediated p38 MAPK signalling in response to cellular stress [34].

The roles of these three proteins in the formation and maintenance of cerebral vessels, the genetic mechanism leading to CCMs and factors that may influence their number and growth are still to be clarified. Actually, PDCD10 is involved in many cellular pathways including apoptosis, cellular proliferation and cell survival/resistance to apoptosis [34]–[39].

**Figure 3:**
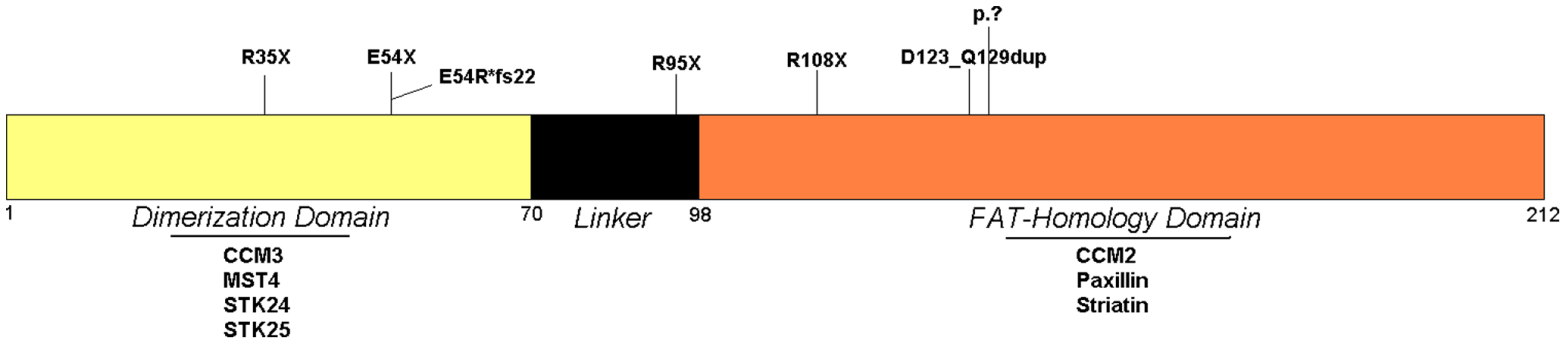
Schematic representation of PDCD10 protein with its domains and the main interactors. Mutations are reported.

PDCD10 protein contains an N-Terminal dimerization Domain and a C-Terminal Focal Adhesion Targeting (FAT) Domain resembling the one of the Focal Adhesion Kinase (FAK) [40], [41] (Fig. 3).

Li et al. demonstrated that the presence of a fully folded CCM3 FAT-Homology Domain is important for the stabilization of the expressed protein in the in vivo setting since an example of a truncation mutation (CCM3-1-117) was found to be poorly expressed [41].

The protein can homodimerize and heterodimerize with a variety of proteins including cell adhesion molecule Paxillin [41] and Malcavernin (CCM2) [12], [42] through its FAT Domain. The N-Terminal domain is important for the interaction with *GCKIII* kinases (Germinal Centre Kinase III), a family of protein kinases, and this heterodimerization may be the preferred conformation [42]. In particular, PDCD10-GCKIII signalling facilitates lumen formation by endothelial cells, which is important during the progression of cerebral lesions [43], [44] and these kinases are important for the regulation of apoptosis, cell proliferation, polarity, migration, and cytoskeleton remodelling [42], [43], [45].

Different animal models have been used to draw these conclusions. In mice model, *Pdcd10* is required for the control of venous size and integrity, yet it is not required in the embryonic establishment of circulation as *Ccm2* is. [44]. Other animal models seem to confirm these findings: tracheal tubes of the respiratory system in *Drosophila melanogaster*, lacking Pdcd10, grow and branch normally, but fail to lumenize [44] and inhibition of *Ccm3a/b* in *Zebrafish* leads to dilations of the embryonic cranial vasculature [46]. Similarly, in vitro studies showed that PDCD10-depleted HUVECs cells failed to organize themselves into a lumenized network [44].

Finally, loss of PDCD10 has been reported to increase cell survival and proliferation, possibly through reduced Notch signalling, enhanced VEGF signalling, or increased ERK activity [39], [47], [48].

These literature data show that PDCD10 interacts with a wide variety of proteins through its different domains, in addition to those involved in CCM pathology (KRIT1, Malcavernin), and participates in several different molecular pathways. So mutations affecting the integrity and stability of CCM3 may disrupt not only the ternary complex with the CCMs proteins but also the interactions with the proteins described above, which act in such different pathways. This pleiotropy of PDCD10 may explain the earlier age at onset we observed in patients mutated in this gene compared to those mutated in *KRIT1*/CCM1 or *MGC4607*/CCM2.

Our data appear to indicate that PDCD10 may play a major role in the ternary complex formed by KRIT1/MALCAVERNIN/PDCD10 proteins and in driving the FCCM associated disorder.

A *PDCD10*/CCM3 gene mutation [40], [41] has to be considered a very rare cause of CCMs, since this mutation have been diagnosed to fewer than 100 people worldwide. Herein we describe 11 additional new cases with a mutation in *PDCD10*/CCM3 gene which are negative for *KRIT1*/CCM1 and *MGC4607*/CCM2 genes. Among the eight mutations identified, four changes have been already reported while other four are described here for the first time; furthermore, we also identified *de novo* mutations in four different patients. In our cohort *de novo* mutations seem to be more frequent in *PDCD10*/CCM3 gene, since no *de novo* mutations have been found in *KRIT1*/CCM1 and only a single *de novo* mutation has been identified in *MGC4607*/CCM2 mutated cases [11].

We tried to establish a common geographic origin between the patients, but the mutations distribution turned out to be rather heterogeneous.

The majority of the detected mutations generates a stop codon (R35X, R108X, E54Rfs*22, E54X), that leads to the predicted formation of a truncated protein lacking the original function, however further studies are needed to define the effect on the protein of the two duplications, the complex rearrangement and the loss of the entire allele on the final protein.

We observe that the majority of mutations especially occur in exons five and seven (8/11 CCM cases), with a prevalence of the R35X mutation (5/11 CCM cases). Thus, we suggest that in an analytical procedure it would be appropriate to first investigate these exons.

All the described mutations in *PDCD10*/CCM3 lead to a stop codon, [15], [17], [19], [27]–[31] that cause the loss of a variable portion of the protein, that generally is the C-Terminal FAT-Homology Domain. Only in a single case (352 CCM) a mutation did not lead to a premature stop codon, but to a in frame duplication of six aminoacids in the FAT-Homology Domain instead. Furthermore this patient showed a late onset disease (35 years) with a milder phenotype compared to other cases. We can hypothesize that this mutation leads to the malfunctioning of the protein but preserves its overall structural integrity and its ability to take interactions with its other partners.

Wide phenotypic variability is present among the reported patients, even within those sharing the same mutation and within the same family. Symptoms range from headache, psychomotor retardation, to attention disorder, haemorrhage and seizures. Together with Denier's group findings [28] we observed an earlier onset in symptomatic *PDCD10*/CCM3 mutation carriers compared to symptomatic patients with a mutation in *KRIT1/*CCM1 and *MGC4607/*CCM2 genes. Conversely, we are unable to confirm the association with multiple meningiomas and early onset haemorrhage reported by Riant F. *et al.*, 2013 [5].

The causes of this variability are unknown but are likely associated with other genetic factors, environment or lifestyle (physical exercise and nutrition), as reported by Choquet et al. for a wide cohort of *KRIT1*/CCM1 patients sharing the Common Hispanic Mutation [49].

Functional characterization of the identified mutations, further *in vitro* studies and cellular models may help understand the complex mechanisms through which PDCD10 is involved in the CCMs pathology.

Indeed, the functional heterogeneity of PDCD10 makes it difficult to define its role in the pathogenesis of the disease and further studies on wider cohorts of patients, as well as prospective clinical follow up studies, will help to define whether *PDCD10*/CCM3 mutations are associated to specific clinical features.

## Material and Methods

### Subjects

Clinically affected CCM probands (index patients) were consecutively enrolled on the basis of one of the two following criteria: each proband had at least one affected relative and/or had multiple cerebral cavernous angiomas. Diagnosis was based on brain magnetic resonance imaging (MRI) features and, when possible, post-surgery histopathological analysis findings: 5/11 patients underwent neurosurgical intervention. Detailed clinical and brain MR imaging data were collected for all patients with symptomatic CCM through direct interview and review of medical records. Clinical assessment focused on the occurrence of seizures, cerebral haemorrhage, focal neurological symptoms, and headache. All the analyzed subjects gave written informed consent and they underwent to a review of their medical records, brain MR imaging, and blood sampling for genetic analysis: their medical records were reviewed as well. Niguarda Ca' Granda Ethic Committee approved this study. Subjects with cavernomas seen on MR images were considered affected and those with no abnormalities seen on MR images were considered unaffected; those who did not undergo MR imaging were classified as “unknown”.

### DNA Extraction, Polymerase Chain Reaction and Sequencing

Genomic DNA from each proband and all consenting relatives was extracted from peripheral blood leukocytes using the salting out method [50]. All coding exons and the corresponding intron/exon boundaries of *KRIT1*/CCM1, *MGC4607*/CCM2 and *PDCD10*/CCM3 genes were amplified by PCR with a specific subset of primers described elsewhere [51].

Direct sequence analysis was performed using BigDye Terminator Cycle Sequencing kit Version 1.1 (Applied Biosystem) on 3730 DNA automated analyzer (Applied Biosystem). The nucleotide position of variants present in the coding regions refers to the mRNA sequence (NM_007217) with +1 corresponding to the A of the ATG initiation codon.

The novel mutations were not found upon screening by direct sequencing 300 normal control chromosomes; moreover they were not reported in different online genetic databases of control subjects, such as HGMD [52], NHLBI ESP [53] and the 1000 Genome project [54].

### Multiplex Ligation-Dependent Probe Amplification Assay

Multiplex ligation-dependent probe amplification (MLPA) was performed on patients who were negative for direct sequencing analysis for mutation in *KRIT1*/CCM1, *MGC4607*/CCM2 and *PDCD10*/CCM3 by using two MLPA kits (SALSA MLPA Kits P130 & P131 CCM, MRCHolland). The P130 probe mix contains probes for part of *KRIT1*/CCM1 exons and for all *MGC4607*/CCM2 exons. The P131 probe mix contains probes for the remaining *KRIT1*/CCM1 exons and for all exons of *PDCD10*/CCM3 gene. MLPA was performed according to the protocol supplied, by use of 100 ng of DNA sample per reaction, using FAM labelled primers. Samples were run on a 3730 DNA automated analyzer (Applied Biosystems), and data were analyzed with the GeneMapper software version 4.0 (Applied Biosystems) to size the PCR products and to obtain peak areas.

For the visual inspection, peak heights were compared between the samples and the controls, to find any alteration in relative peak heights within the test sample. For the normalized peak area calculations, each peak area was normalized by dividing the individual peak area by the total peak area of all peaks for that sample. See Penco et al., 2009 for details [24].

### Short Tandem Repeat Multiplex Assay

STR (Short Tandem Repeat) multiplex assay was performed by using the AmpFlSTR Identifiler Kit (Applied Biosystems) according to the manufacturer's instructions. The kit has been designed to amplify 15 tetranucleotide repeat loci and the amelogenin gender-determining marker in a single PCR amplification; a five-dye fluorescent system was used for automated DNA fragment analysis.

Samples were run on a 3730 DNA automated analyzer (Applied Biosystems), and data were analyzed with the Gene Mapper software version 4.0 (Applied Biosystems); allele peaks were interpreted when the peak heights were greater than or equal to 50 relative fluorescence units.

## References

[1] Gomori JM, Grossman RI, Goldberg HI, Hackney DB, Zimmerman RA, et al (1986) Occult cerebral vascular malformations: high-field MR imaging. Radiology 158: 707–713. 3945744 10.1148/radiology.158.3.3945744

[2] Labauge P, Denier C, Bergametti F, Tournier-Lasserve E (2007) Genetics of cavernous angiomas. Lancet Neurol 6: 237–244. 17303530 10.1016/S1474-4422(07)70053-4

[3] Wilkins RH (1985) Natural history of intracranial vascular malformations: a review. Neurosurgery 16: 421–430 10.1227/00006123-198503000-00026 3885072

[4] Porter PJ, Willinsky RA, Harper W, Wallace MC (1997) Cerebral cavernous malformations: natural history and prognosis after clinical deterioration with or without hemorrhage. J Neurosurg 87: 190–197 10.3171/jns.1997.87.2.0190 9254081

[5] Riant F, Bergametti F, Fournier H-D, Chapon F, Michalak-Provost S, et al (2013) CCM3 Mutations Are Associated with Early-Onset Cerebral Hemorrhage and Multiple Meningiomas. Mol Syndromol 4: 165–172 Available: http://www.pubmedcentral.nih.gov/articlerender.fcgi?artid=3666455&tool=pmcentrez&rendertype=abstract. Accessed 21 July 2014 23801932 10.1159/000350042 PMC3666455

[6] Rigamonti D, Hadley MN, Drayer BP, Johnson PC, Hoenig-Rigamonti K, et al (1988) Cerebral cavernous malformations. Incidence and familial occurrence. N Engl J Med 319: 343–347 10.1056/NEJM198808113190605 3393196

[7] Bicknell JM, Carlow TJ, Kornfeld M, Stovring J, Turner P (1978) Familial cavernous angiomas. Arch Neurol 35: 746–749 10.1212/01.WNL.0000142982.80308.D9 718473

[8] Coban A, Gurses C, Bilgic B, Sencer S, Karasu A, et al (2008) Sporadic multiple cerebral cavernomatosis: report of a case and review of literature. Neurologist 14: 46–49 10.1097/NRL.0b013e31813e343f 18195658

[9] Gaetzner S, Stahl S, Surucu O, Schaafhausen A, Halliger-Keller B, et al (2007) CCM1 gene deletion identified by MLPA in cerebral cavernous malformation. Neurosurg Rev 30: 155–159. 17187287 10.1007/s10143-006-0057-1

[10] Riant F, Bergametti F, Ayrignac X, Boulday G, Tournier-Lasserve E (2010) Recent insights into cerebral cavernous malformations: the molecular genetics of CCM. FEBS J 277: 1070–1075 10.1111/j.1742-4658.2009.07535.x 20096038

[11] Mosca L, Pileggi S, Avemaria F, Tarlarini C, Cigoli MS, et al (2012) De novo MGC4607 gene heterozygous missense variants in a child with multiple cerebral cavernous malformations. J Mol Neurosci 47: 475–480. 22415356 10.1007/s12031-012-9741-5

[12] Laberge-le Couteulx S, Jung HH, Labauge P, Houtteville JP, Lescoat C, et al (1999) Truncating mutations in CCM1, encoding KRIT1, cause hereditary cavernous angiomas. Nat Genet 23: 189–193. 10508515 10.1038/13815

[13] Sahoo T, Johnson EW, Thomas JW, Kuehl PM, Jones TL, et al (1999) Mutations in the gene encoding KRIT1, a Krev-1/rap1a binding protein, cause cerebral cavernous malformations (CCM1). Hum Mol Genet 8: 2325–2333 Available: http://www.ncbi.nlm.nih.gov/pubmed/10545614. Accessed 10 July 2014 10545614 10.1093/hmg/8.12.2325

[14] Denier C, Goutagny S, Labauge P, Krivosic V, Arnoult M, et al (2004) Mutations within the MGC4607 gene cause cerebral cavernous malformations. Am J Hum Genet 74: 326–337 Available: http://www.pubmedcentral.nih.gov/articlerender.fcgi?artid=1181930&tool=pmcentrez&rendertype=abstract. Accessed 10 July 2014 14740320 10.1086/381718 PMC1181930

[15] Liquori CL, Penco S, Gault J, Leedom TP, Tassi L, et al (2008) Different spectra of genomic deletions within the CCM genes between Italian and American CCM patient cohorts. Neurogenetics 9: 25–31 Available: http://www.ncbi.nlm.nih.gov/pubmed/18060436. Accessed 21 July 2014 18060436 10.1007/s10048-007-0109-x

[16] Bergametti F, Denier C, Labauge P, Arnoult M, Boetto S, et al (2005) Mutations within the programmed cell death 10 gene cause cerebral cavernous malformations. Am J Hum Genet 76: 42–51 Available: http://www.ncbi.nlm.nih.gov/entrez/query.fcgi?cmd=Retrieve&db=PubMed&dopt=Citation&list_uids=15543491 15543491 10.1086/426952 PMC1196432

[17] Guclu B, Ozturk AK, Pricola KL, Bilguvar K, Shin D, et al (2005) Mutations in apoptosis-related gene, PDCD10, cause cerebral cavernous malformation 3. Neurosurgery 57: 1008–1012. 16284570 10.1227/01.neu.0000180811.56157.e1

[18] Marini V, Ferrera L, Dorcaratto A, Viale G, Origone P, et al (2003) Identification of a novel KRIT1 mutation in an Italian family with cerebral cavernous malformation by the protein truncation test. J Neurol Sci 212: 75–78 Available: http://www.ncbi.nlm.nih.gov/pubmed/12810002. Accessed 21 July 2014 12810002 10.1016/s0022-510x(03)00108-4

[19] Liquori CL, Berg MJ, Squitieri F, Ottenbacher M, Sorlie M, et al (2006) Low frequency of PDCD10 mutations in a panel of CCM3 probands: potential for a fourth CCM locus. Hum Mutat 27: 118. 10.1002/humu.9389 16329096

[20] Battistini S, Rocchi R, Cerase A, Citterio A, Tassi L, et al (2007) Clinical, magnetic resonance imaging, and genetic study of 5 Italian families with cerebral cavernous malformation. Arch Neurol 64: 843–848 Available: http://www.ncbi.nlm.nih.gov/entrez/query.fcgi?cmd=Retrieve&db=PubMed&dopt=Citation&list_uids=17562932 17562932 10.1001/archneur.64.6.843

[21] Gianfrancesco F, Cannella M, Martino T, Maglione V, Esposito T, et al (2007) Highly variable penetrance in subjects affected with cavernous cerebral angiomas (CCM) carrying novel CCM1 and CCM2 mutations. Am J Med Genet B Neuropsychiatr Genet 144B: 691–695 Available: http://www.ncbi.nlm.nih.gov/pubmed/17440989. Accessed 21 July 2014 17440989 10.1002/ajmg.b.30381

[22] Guarnieri V, Muscarella LA, Amoroso R, Quattrone A, Abate ME, et al (2007) Identification of two novel mutations and of a novel critical region in the KRIT1 gene. Neurogenetics 8: 29–37. 17043900 10.1007/s10048-006-0056-y

[23] Nannucci S, Pescini F, Poggesi A, Ciolli L, Patrosso MC, et al (2009) Familial cerebral cavernous malformation: Report of a further Italian family. Neurol Sci 30: 143–147. 19184323 10.1007/s10072-009-0020-3

[24] Penco S, Ratti R, Bianchi E, Citterio A, Patrosso MC, et al (2009) Molecular screening test in familial forms of cerebral cavernous malformation: the impact of the Multiplex Ligation-dependent Probe Amplification approach. J Neurosurg 110: 929–934 10.3171/2008.8.17640 19199464

[25] Muscarella LA, Guarnieri V, Coco M, Belli S, Parrella P, et al.. (2010) Small deletion at the 7q21.2 locus in a CCM family detected by Real-Time Quantitative PCR. J Biomed Biotechnol 2010. 10.1155/2010/854737 PMC2926733 20798775

[26] Pileggi S, Buscone S, Ricci C, Patrosso MC, Marocchi A, et al (2010) Genetic variations within KRIT1/CCM1, MGC4607/CCM2 and PDCD10/CCM3 in a large italian family harbouring a krit1/CCM1 mutation. J Mol Neurosci 42: 235–242. 20419355 10.1007/s12031-010-9360-y

[27] Verlaan DJ, Roussel J, Laurent SB, Elger CE, Siegel AM, et al (2005) CCM3 mutations are uncommon in cerebral cavernous malformations. Neurology 65: 1982–1983 Available: http://www.ncbi.nlm.nih.gov/pubmed/16380626. Accessed 21 July 2014 16380626 10.1212/01.wnl.0000188903.75144.49

[28] Denier C, Labauge P, Bergametti F, Marchelli F, Riant F, et al (2006) Genotype-phenotype correlations in cerebral cavernous malformations patients. Ann Neurol 60: 550–556 Available: http://www.ncbi.nlm.nih.gov/entrez/query.fcgi?cmd=Retrieve&db=PubMed&dopt=Citation&list_uids=17041941 17041941 10.1002/ana.20947

[29] Gault J, Sain S, Hu LJ, Awad IA (2006) Spectrum of genotype and clinical manifestations in cerebral cavernous malformations. Neurosurgery 59: 1278–1284. 17277691 10.1227/01.NEU.0000249188.38409.03

[30] Lee S-T, Choi K-W, Yeo H-T, Kim J-W, Ki C-S, et al (2008) Identification of an Arg35X mutation in the PDCD10 gene in a patient with cerebral and multiple spinal cavernous malformations. J Neurol Sci 267: 177–181 Available: http://www.ncbi.nlm.nih.gov/pubmed/18035376. Accessed 21 July 2014 18035376 10.1016/j.jns.2007.10.018

[31] Choe C, Riant F, Gerloff C, Tournier-Lasserve E, Orth M (2010) Multiple cerebral cavernous malformations and a novel CCM3 germline deletion in a German family. J Neurol 257: 2097–2098 Available: http://www.ncbi.nlm.nih.gov/pubmed/20623299. Accessed 21 July 2014 20623299 10.1007/s00415-010-5648-7

[32] Wildeman M, Van Ophuizen E, Den Dunnen JT, Taschner PEM (2008) Improving sequence variant descriptions in mutation databases and literature using the mutalyzer sequence variation nomenclature checker. Hum Mutat 29: 6–13. 18000842 10.1002/humu.20654

[33] Schwarz JM, Rödelsperger C, Schuelke M, Seelow D (2010) MutationTaster evaluates disease-causing potential of sequence alterations. Nat Methods 7: 575–576 Available: http://www.ncbi.nlm.nih.gov/pubmed/20676075. Accessed 11 July 2014 20676075 10.1038/nmeth0810-575

[34] Voss K, Stahl S, Schleider E, Ullrich S, Nickel J, et al (2007) CCM3 interacts with CCM2 indicating common pathogenesis for cerebral cavernous malformations. Neurogenetics 8: 249–256. 17657516 10.1007/s10048-007-0098-9

[35] Ma X, Zhao H, Shan J, Long F, Chen Y, et al (2007) PDCD10 interacts with Ste20-related kinase MST4 to promote cell growth and transformation via modulation of the ERK pathway. Mol Biol Cell 18: 1965–1978. 17360971 10.1091/mbc.E06-07-0608 PMC1877091

[36] Chen L, Tanriover G, Yano H, Friedlander R, Louvi A, et al (2009) Apoptotic functions of PDCD10/CCM3, the gene mutated in cerebral cavernous malformation 3. Stroke 40: 1474–1481 Available: http://www.pubmedcentral.nih.gov/articlerender.fcgi?artid=2709460&tool=pmcentrez&rendertype=abstract. Accessed 21 July 2014 19246713 10.1161/STROKEAHA.108.527135 PMC2709460

[37] Goudreault M, D'Ambrosio LM, Kean MJ, Mullin MJ, Larsen BG, et al (2009) A PP2A phosphatase high density interaction network identifies a novel striatin-interacting phosphatase and kinase complex linked to the cerebral cavernous malformation 3 (CCM3) protein. Mol Cell Proteomics 8: 157–171 Available: http://www.pubmedcentral.nih.gov/articlerender.fcgi?artid=2621004&tool=pmcentrez&rendertype=abstract. Accessed 18 July 2014 18782753 10.1074/mcp.M800266-MCP200 PMC2621004

[38] Faurobert E, Albiges-Rizo C (2010) Recent insights into cerebral cavernous malformations: A complex jigsaw puzzle under construction. FEBS J 277: 1084–1096. 20096036 10.1111/j.1742-4658.2009.07537.x PMC3076058

[39] Louvi A, Chen L, Two AM, Zhang H, Min W, et al (2011) Loss of cerebral cavernous malformation 3 (Ccm3) in neuroglia leads to CCM and vascular pathology. Proc Natl Acad Sci U S A 108: 3737–3742. 21321212 10.1073/pnas.1012617108 PMC3048113

[40] Ding J, Wang X, Li D-F, Hu Y, Zhang Y, et al (2010) Crystal structure of human programmed cell death 10 complexed with inositol-(1,3,4,5)-tetrakisphosphate: a novel adaptor protein involved in human cerebral cavernous malformation. Biochem Biophys Res Commun 399: 587–592 10.1016/j.bbrc.2010.07.119 20682288

[41] Li X, Zhang R, Zhang H, He Y, Ji W, et al (2010) Crystal structure of CCM3, a cerebral cavernous malformation protein critical for vascular integrity. J Biol Chem 285: 24099–24107. 20489202 10.1074/jbc.M110.128470 PMC2911348

[42] Ceccarelli DF, Laister RC, Mulligan VK, Kean MJ, Goudreault M, et al (2011) CCM3/PDCD10 heterodimerizes with germinal center kinase III (GCKIII) proteins using a mechanism analogous to CCM3 homodimerization. J Biol Chem 286: 25056–25064 Available: http://www.pubmedcentral.nih.gov/articlerender.fcgi?artid=3137079&tool=pmcentrez&rendertype=abstract. Accessed 21 July 2014 21561863 10.1074/jbc.M110.213777 PMC3137079

[43] Zheng X, Xu C, Di Lorenzo A, Kleaveland B, Zou Z, et al (2010) CCM3 signaling through sterile 20-like kinases plays an essential role during zebrafish cardiovascular development and cerebral cavernous malformations. J Clin Invest 120: 2795–2804. 20592472 10.1172/JCI39679 PMC2912181

[44] Chan AC, Drakos SG, Ruiz OE, Smith ACH, Gibson CC, et al (2011) Mutations in 2 distinct genetic pathways result in cerebral cavernous malformations in mice. J Clin Invest 121: 1871–1881. 21490399 10.1172/JCI44393 PMC3083782

[45] Fidalgo M, Fraile M, Pires A, Force T, Pombo C, et al (2010) CCM3/PDCD10 stabilizes GCKIII proteins to promote Golgi assembly and cell orientation. J Cell Sci 123: 1274–1284 Available: http://www.ncbi.nlm.nih.gov/pubmed/20332113. Accessed 17 July 2014 20332113 10.1242/jcs.061341

[46] Yoruk B, Gillers BS, Chi NC, Scott IC (2012) Ccm3 functions in a manner distinct from Ccm1 and Ccm2 in a zebrafish model of CCM vascular disease. Dev Biol 362: 121–131 Available: http://www.ncbi.nlm.nih.gov/pubmed/22182521 Accessed 23 July 2014 22182521 10.1016/j.ydbio.2011.12.006

[47] Zhu Y, Wu Q, Xu J-F, Miller D, Sandalcioglu IE, et al (2010) Differential angiogenesis function of CCM2 and CCM3 in cerebral cavernous malformations. Neurosurg Focus 29: E1 10.3171/2010.5.FOCUS1090 20809750

[48] You C, Erol Sandalcioglu I, Dammann P, Felbor U, Sure U, et al (2013) Loss of CCM3 impairs DLL4-Notch signalling: Implication in endothelial angiogenesis and in inherited cerebral cavernous malformations. J Cell Mol Med 17: 407–418. 23388056 10.1111/jcmm.12022 PMC3823022

[49] Choquet H, Nelson J, Pawlikowska L, McCulloch CE, Akers A, et al (2014) Association of cardiovascular risk factors with disease severity in cerebral cavernous malformation type 1 subjects with the common hispanic mutation. Cerebrovasc Dis 37: 57–63. 24401931 10.1159/000356839 PMC3995158

[50] Miller SA, Dykes DD, Polesky HF (1988) A simple salting out procedure for extracting DNA from human nucleated cells. Nucleic Acids Res 16: 1215. 3344216 10.1093/nar/16.3.1215 PMC334765

[51] Cavé-Riant F, Denier C, Labauge P, Cécillon M, Maciazek J, et al (2002) Spectrum and expression analysis of KRIT1 mutations in 121 consecutive and unrelated patients with Cerebral Cavernous Malformations. Eur J Hum Genet 10: 733–740 Available: http://www.ncbi.nlm.nih.gov/pubmed/12404106 Accessed 10 July 2014 12404106 10.1038/sj.ejhg.5200870

[52] The Human Gene Mutation Database at the Institute of Medical Genetics in Cardiff. (n.d.). Available: http://www.hgmd.cf.ac.uk/ac. Accessed 27 September 2013.

[53] The National Heart, Lung, and Blood Institute Exome Sequencing Project. (n.d.). Available: http://evs.gs.washington.edu/EVS/. Accessed 27 September 2013.

[54] The 1000 Genomes Project. (n.d.). Available: http://www.1000genomes.org/. Accessed 27 September 2013.

