# Correction: *PDCD10* Gene Mutations in Multiple Cerebral Cavernous Malformations

**DOI:** 10.1371/journal.pone.0123486

**Published:** 2015-04-07

**Authors:** The PLOS ONE Staff

There are errors in affiliation 8 for author Lilia Volpi. Affiliation 8 should be: Neurology Unit, IRCCS Institute of Neurological Sciences, AUSL Bologna–Bellaria Pizzardi Hospital, Bologna, Italy.
